# The effectiveness of *Grief-Help*, a cognitive behavioural treatment for prolonged grief in children: study protocol for a randomised controlled trial

**DOI:** 10.1186/1745-6215-14-395

**Published:** 2013-11-20

**Authors:** Mariken Spuij, Peter Prinzie, Maja Dekovic, Jan van den Bout, Paul A Boelen

**Affiliations:** 1 Department of Child and Adolescent Studies, Utrecht University, PO Box 80140, Utrecht, TC 3508, The Netherlands; 2 Department of Clinical and Health Psychology, Utrecht University, PO Box 80140, Utrecht, TC 3508, The Netherlands

**Keywords:** Adolescents, Children, Cognitive-behavioural treatment, Prolonged grief disorder, Randomised controlled trial

## Abstract

**Background:**

There is growing recognition of a syndrome of disturbed grief referred to as prolonged grief disorder (PGD). PGD is mostly studied in adults, but clinically significant PGD symptoms have also been observed in children and adolescents. Yet, to date no effective treatment for childhood PGD exists. The aims of this study are: (1) to investigate the effectiveness of *Grief-Help*, a nine-session cognitive-behavioural treatment for childhood PGD, combined with five sessions of parental counselling, immediately after the treatment and at three, six and twelve months follow-up; (2) to examine tentative mediators of the effects of *Grief-Help,* (i.e., maladaptive cognitions and behaviours and positive parenting), and (3) to determine whether demographic variables, child personality, as well as symptoms of PGD, anxiety, and depression in parents moderate the treatment effectiveness.

**Methods/Design:**

We will conduct a Randomised Controlled Trial (RCT) in which 160 children and adolescents aged 8–18 years are randomly allocated to cognitive behavioural *Grief-Help* or to a *supportive counselling* intervention; both treatments are combined with five sessions of parental counselling. We will recruit participants from clinics for mental health in the Netherlands. The primary outcome measure will be the severity of Prolonged Grief Disorder symptoms according to the Inventory of Prolonged Grief for Children (IPG-C). Secondary outcomes will include PTSD, depression and parent-rated internalizing and externalizing problems. Mediators like positive parenting and maladaptive cognitions and behaviours will be identified. We will also examine possible moderators including demographic variables (e.g. time since loss, cause of death), psychopathology symptoms in parents (PGD, anxiety and depression) and child personality. Assessments will take place in both groups at baseline, after the treatment-phase and three, six and twelve months after the post-treatment assessment.

**Discussion:**

We aim to contribute to the improvement of mental health care for children and adolescents suffering from loss. By comparing *Grief-Help* with *supportive counselling*, and by investigating mediators and moderators of its effectiveness we hope to provide new insights in the effects of interventions for bereaved children, and their mechanisms of change.

**Trial registration:**

Netherlands Trial Register NTR3854

## Background

The death of a loved one in childhood and adolescence is associated with increased emotional problems, including elevated depression, anxiety and posttraumatic stress, as well as somatic complaints and behavioural problems [1,2]. From among all children who experience such a loss, an estimated 5% to 10% go on to experience clinically significant psychiatric problems. Such problems include major depression, posttraumatic stress disorder (PTSD) and prolonged grief disorder (PGD) [3,4].

PGD encompasses several symptoms, including separation distress, preoccupation with thoughts about the lost person, a sense of purposelessness about the future, numbness, bitterness, difficulty accepting the loss and difficulty moving on with life without the lost person [5,6]. Empirical studies have shown that PGD symptoms can be reliably assessed in children and adolescents [7]. PGD symptoms can be distinguished from normal grief, depression and anxiety, including PTSD, and are associated with significant concomitant internalizing and externalizing problems [7-10].

Few effective interventions for bereaved children and adolescents are available. On the basis of a meta-analysis of 13 controlled studies examining the effectiveness of bereavement interventions with children, Currier *et al*. [11] concluded that interventions available at the time were not more useful than undergoing no intervention. Indeed, a number of controlled studies have been conducted on the treatment of bereaved children, including studies examining family interventions for bereaved children [12], music therapy in groups [13], group therapy for children [14] and group therapy for children bereaved by the suicide of a relative [15]. These studies are limited by the fact that they did not articulate the theoretical basis of the intervention tested [11], focused on generic indices of distress rather than on symptoms of grief [12,15] or did not randomly allocate participants to treatment and control groups [13,14].

In the past decade, several promising lines of research have greatly advanced our understanding of bereavement interventions for children. The first and most extensive line of research concerns the family bereavement program (FBP), developed by Sandler and colleagues [16,17]. The FBP is a group-based program that targets family-level variables (for example, parenting skills) and child-level variables (for example, coping skills) that promote resilience. The FBP was found to reduce immediate and long-term emotional problems in children confronted with parental loss. A second promising intervention is the trauma and grief component therapy (TGCT) developed by Layne *et al*. [18]. TGCT is a group treatment for adolescents confronted with loss in the context of a civil war. This treatment proved to be effective in terms of reducing grief, depression and anxiety symptoms [2,18]. A third important line of research concerns the work of Cohen and colleagues [19,20] on cognitive-behavioural therapy for childhood traumatic grief (CBT-CTG). This treatment approach explicitly focuses on the alleviation of the emotional condition termed *childhood traumatic grief*, which is defined as a combination of traumatic and grief stress reaction, among children exposed to deaths that occurred under traumatic circumstances (for example, motor vehicle accidents, suicide, homicide). Two uncontrolled studies showed that children who underwent CBT-CTG reported significant improvement in CTG and PTSD symptoms [19,20].

Notwithstanding the importance of these three research lines, they still leave room for further study and refinement of treatment options for children confronted with the death of a loved one. For instance, the FBP and TGCT are limited to a group-based format, which may yield practical problems; for example, clients may have to wait until there are enough children for a group and may be less effective because it is less well-adjusted to an individual child’s circumstances. In addition, all three approaches are limited by their focus on restricted groups, such as parentally bereaved children (FBP) or children exposed to traumatic deaths (TGCT and CBT-CTG), making these approaches less suitable for use with other groups of bereaved children. The impact of these treatments on PGD symptoms, as currently defined [5,6], is unknown.

Given the need for effective therapy for PGD symptoms in children and adolescents, we developed a nine-session protocolized cognitive-behavioural treatment that is administered in combination with five sessions of parental counselling. This treatment is called *Grief-Help*. It is based on a cognitive-behavioural model of processes that interfere with adjustment to loss. Two pilot studies of this treatment have been done.

The first was a multiple-baseline study of six bereaved children and adolescents, which showed that the intervention coincided with reductions in symptoms of PGD, depression, PTSD and (parent-rated) internalizing and externalizing problems [21]. The intervention proved to be feasible, as both children and parents evaluated the treatment positively. That is, all participating children and parents gave favourable scores regarding their satisfaction with each session, the contact with their therapist and the information they received, attesting to the feasibility of this treatment approach. Results showed that after treatment there were reductions in symptoms of PGD, depression, posttraumatic stress and parent-rated internalizing and externalizing problems. Averaged across the six participants, reductions in scores on the outcome measures were all statistically significant, and all pretreatment to posttreatment effect sizes were large (Cohen’s *d* > 0.8).

The second pilot study was an open trial conducted with ten children and adolescents [22]. We conducted this study to evaluate the potential effectiveness of Grief-Help therapy among children confronted with losses other than the loss of a parent or sibling and to investigate whether the program is effective when the loss occurred more than 12 months prior to initiation of treatment. In this study, patients significantly improved from pretreatment to posttreatment, with large improvements observed in self-rated PGD and bereavement-related posttraumatic stress (effect size (ES) > 0.8) and small to moderate improvements in depression and parent-rated internalizing and externalizing problems (0.2 < ES < 0.8). Additional analyses focused on predictors of treatment outcomes suggested that Grief-Help therapy might be less effective for children and adolescents who are further removed in time from the loss and for those confronted with loss due to suicide. Taken together, Grief-Help therapy appears to be a promising treatment, and controlled evaluation is clearly indicated.

### Trial objective

This randomised controlled trial seeks to examine the effect of cognitive-behavioural Grief-Help therapy for children with emotional problems following the death of a loved one. Participants are randomly assigned to one of two treatment conditions: (1) cognitive-behavioural Grief-Help therapy combined with parental support or (2) a control treatment consisting of nondirective supportive counselling combined with parental support. Participants are asked to complete questionnaires before and after treatment and at three follow-up assessment points.

This treatment trail has three goals. First, we want to compare the effects of cognitive behavioural Grief-Help therapy with the effects of supportive counselling by measuring the reduction of PGD symptoms and other emotional problems, including depression and PTSD. Our second goal is to gain knowledge about variables that are expected to mediate the effects of Grief-Help therapy, such as maladaptive cognition, avoidance behaviours and positive parenting (warmth, involvement and autonomy-granting). We also want to generate knowledge about variables that moderate the effectiveness of Grief-Help therapy.

We hypothesize that the Grief-Help group will show a greater reduction of PGD symptoms and other emotional problems (for example, depression, PTSD symptoms) than the supportive counselling group immediately after treatment and at each follow-up point (three, six and twelve months later). Furthermore, we expect that this reduction will be mediated by a change in maladaptive cognitions and behaviours as well as by increases in positive parenting. We consider the following factors to be possible moderators: demographic variables, severity of symptoms before treatment, time since loss, cause of death, child personality and psychopathology symptoms in parents. We will use state-of-the art statistical techniques to analyse temporality, causality and mechanisms of change.

## Method/Design

### Study design

**Figure 1:**
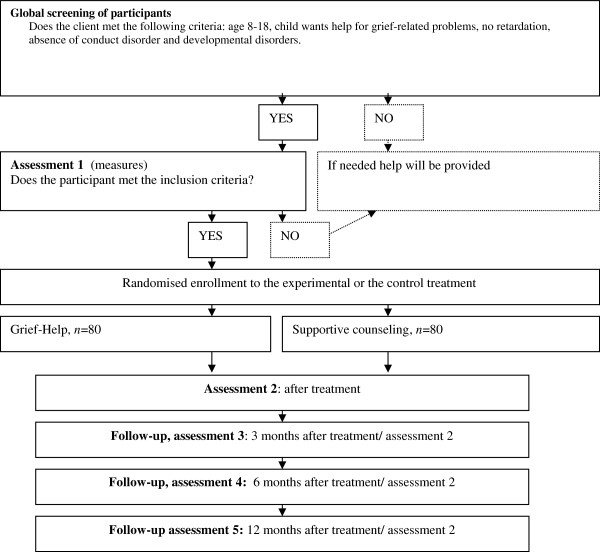
Diagram showing flow of participants through the study.

We will conduct a randomised controlled trial with two intervention groups: Grief-Help therapy versus supportive counselling (Figure 1). Ethical approval was granted by an independent medical ethics committee (Central Committee on Research Involving Human Subjects NL30528.041.09).

### Participants

Participants will be bereaved children and adolescents (and their parents) who apply for help in outpatient mental health care clinics in The Netherlands. The following inclusion criteria will be used: (1) ages 8 through 18 years, (2) loss of a close relative, (3) symptoms of PGD as the primary problem and reason for seeking therapy, (4) absence of mental retardation, (5) absence of severe conduct disorder and developmental disorders, (6) no concurrent psychological or psychopharmacological treatment and (7) no current substance abuse or dependence, no psychotic symptoms and no severe depression with risk of suicide in participating children or their parents.

### Procedure and randomisation

Potentially eligible participants (and their parents) referred to the participating clinics by their general practitioners will receive oral and written information about the research program. Surviving parents’ consent will be obtained for their children to participate. Assent or consent will be obtained from the children, depending on their age. Children will then complete baseline measures (assessment 1 (A1)) in the presence of a therapist. Parents will also complete baseline measures at A1.

Randomisation will take place after informed consent is obtained and after completion of the baseline measures (A1). Participants will be randomly allocated to one of two treatment conditions. Both treatments will be introduced to participants as potentially useful interventions for bereaved children. Four follow-up assessments will be conducted: directly after completion of the treatment (A2) and three, six and twelve months later (A3, A4 and A5, respectively).

### Measures

All measures, time points and informants are summarized in the assessment schedule (Table 1).

**Table 1:** **Assessment schedule**^**a**^

**Variable**	**Concept**	**Measure**	**Informant**	**Assessment**	**Goal**
Primary outcome measure	PGD symptoms	IPG-C	Child	A1, A2, A3, A4, A5	1, 2, 3
Secondary outcome measures	PTSS symptoms	CPSS	Parent	A1, A2, A3, A4, A5	1, 2, 3
	Depression symptoms	CDI	Child	A1, A2, A3, A4, A5	1, 2, 3
	Behaviour problems	CBCL	Child	A1, A2, A3, A4, A5	1, 2, 3
Mediator	Positive parenting	PPQ, MFP	Parent and child	A1, A2, A3, A4, A5	2
	Maladaptive cognitions and behaviours	GCQ-C-II, GBQ-C-II	Child	A1, A2, A3, A4, A5	2
Moderator	Demographic variables	Demographic questions	Parent and child	A1	3
	PGD symptoms (parents)	ICG-R	Parent	A1	3
	Anxiety and depression (parents)	HADS	Parent	A1	3
	Child personality	H*i*PIC	Parent	A1	3

^a^CBCL = Child Behavior Checklist, CDI = Children’s Depression Inventory, CPSS = Child Posttraumatic Stress Disorder Symptom Scale, GBQ-C = Grief Behavior Questionnaire for Children, GCI-C = Grief Cognition Questionnaire for Children, HADS = Hospital Anxiety and Depression Scale, H*i*PIC = Hierarchical Personality Inventory for Children, ICG-R = Inventory of Prolonged Grief–Revised, IPG-C = Inventory of Prolonged Grief for Children, MFP = Mother-Father-Peer Scale, PPQ = Parenting Practices Questionnaire.

### Primary outcome measure

#### Inventory of prolonged grief for children

The Inventory of Prolonged Grief for Children (IPG-C) is a 30-item measure of PGD symptoms. It is an adapted version of the Inventory of Complicated Grief developed to assess adult PGD [23] that taps all symptoms listed in the PGD criteria proposed for the *Diagnostic and Statistical Manual of Mental Disorders, Fifth Edition* (DSM-5),and the *International Classification of Diseases, 11th Revision* (ICD-11) [5], as well as several additional markers of dysfunctional grief. The IPG-C rates symptom frequency in the past month on three-point scales ranging from 1 (almost never) to 3 (always). The measure has good internal consistency, stability and concurrent validity (for example, high correlations with other measures of grief) [10].

### Secondary outcome measures

#### Child PTSD symptom scale

The Child PTSD Symptom Scale (CPSS) is a 17-item questionnaire for the assessment of PTSD symptoms, as defined in the DSM-IV, constructed by Foa *et al*. [24]. Respondents rate the occurrence of symptoms on four-point scales ranging from 0 (not at all/only once a week) to 3 (almost always/five or more times a week). The index event is defined as “the death of your loved one”. Research has shown that the CPSS has good reliability and convergent and discriminant validity [24-26].

#### Children’s depression inventory

The Children’s Depression Inventory (CDI) is a well-validated 27-item measure of depression symptoms [27-29]. Each item contains three statements representing depressive symptoms at increasing levels of severity, from among which respondents select the one statement that best describes how he or she felt during the preceding week.

#### Child behavior checklist

The Child Behavior Checklist (CBCL) [30] is a 118-item measure of emotional and behavioural problems of children 6 to 18 years of age that is completed by parents. Items are rated on three-point scales with the anchors being 0 (not true) and 2 (very true/often true). Scores can be used to obtain indices of internalizing problems and externalizing problems. The summed score of all items represents a Total Problem score. The psychometric properties of the original version [30] and Dutch version [31] are adequate.

### Potential mediators

#### Positive parenting

##### Parenting practices questionnaire

Two scales of the Parenting Practices Questionnaire (PPQ) [32] are used: warmth and involvement (11 items) and reasoning/induction (7 items). According to Locke and Prinz [33], the PPQ has adequate psychometric characteristics. The items are rated on a five-point scale ranging from 1 (never) to 5 (always).

##### Mother-father-peer scale

The Mother-Father-Peer Scale (MFP) is administered to assess psychological autonomy-granting, an aspect of positive parenting [34]. Only the mother and father parts of the scale are included. Because of time constraints, a slightly shorter version of the scale from the original inventory will be used (nine of the original thirteen items). Using a Likert scale ranging from 1 (not at all) to 5 (very much), each of the parents fills out the scales with regard to his or her relationship with their child. The inventory has good reliability and has been validated against several other measures of parenting [35].

### Maladaptive cognitions and behaviours

#### Grief cognition questionnaire for children

The Grief Cognition Questionnaire for Children (GCQ-C) is a 20-item measure of maladaptive grief cognitions and is based on the Grief Cognitions Questionnaire for adults [36]. The CGQ-C rates the frequency of maladaptive cognitions during the past 2 weeks on four-point scales ranging from 1 (never) to 4 (very often). The internal consistency, temporal stability and concurrent and construct validity of the questionnaire are adequate (M Spuij, PP Prinzie and PA Boelen, unpublished observations).

#### Grief behaviour questionnaire for children

The Grief Behaviour Questionnaire for Children (GBQ-C) was specifically designed for this study to assess strategies to avoid confrontation with the reality of the loss (called “anxious avoidance”) and a tendency to refrain from activities that could foster adjustment (called “depressive avoidance”). Its 12 items are based on items from the Depressive and Anxious Avoidance in Prolonged Grief Questionnaire (DAAPGQ) developed by Boelen, Van den Bout and colleagues [37,38].

### Potential moderators

#### Hospital anxiety and depression scale

The Hospital Anxiety and Depression Scale (HADS) [39] is a 14-item measure of anxiety and depression symptoms in adults. The Dutch version of the HADS has been validated [40]. Items are rated on a four-point Likert scale, with higher ratings indicating higher states of anxiety or depression.

#### Inventory of complicated grief–revised

The Inventory of Complicated Grief–Revised (ICG-R) was developed by Prigerson and Jacobs [23] as a 30-item measure of PGD symptoms in adults. The Dutch version of the measure has good internal consistency, stability and concurrent validity [41]. Respondents rate the occurrence of these symptoms during the past month on five-point scales ranging from “never” to “all the time”.

#### Hierarchical personality inventory for children

The Hierarchical Personality Inventory for Children (H*i*PIC) is a 144-item personality inventory that assesses individual differences between children within the framework of the Big Five, which is completed by parents. Dimensions that can be identified are extraversion (32 items), benevolence (40 items), conscientiousness (32 items), emotional stability (16 items) and imagination (24 items). It has been shown to have high convergent and discriminant validity as well as temporal stability [42,43]. The items are scored on a five-point scale, ranging from 1 (hardly characteristic) to 5 (very characteristic).

### Other information

Demographic information will be assessed at baseline. Treatment adherence will be measured in terms of the number of sessions attended and completion of homework assignments. Adherence to both treatment protocols will be promoted by regular meetings with the therapist. Therapy sessions will be audiotaped, and randomly selected tapes will be discussed to ensure that therapists adhere to the protocols.

### Sample size

Our sample size calculation is based on the conventional significance (α) and power (1 – β) levels of 0.05 and 0.80, respectively, planning one-sided testing. The sample size of this study is based on the expected difference on the primary outcome variable (that is, PGD symptoms) between the two conditions. On the basis of a power of 0.80, an α of 0.05 and an expected dropout percentage of 20%, we will need 80 participants for each condition to show an effect size of 0.50 [44]. Therefore, we have determined the total sample size to be 160.

### Experimental and control treatment

Clients will receive nine individual 45-minute sessions of therapy for childhood PGD, which are planned to occur once every 1 or 2 weeks. Five 45-minute sessions with one or two of the parents will be planned to be conducted in parallel with these nine sessions. Both treatments will follow the format described by Spuij *et al*. [21]. Treatments will be conducted by licensed (post–master’s degree level) therapists.

### Grief-help therapy

Grief-Help therapy for childhood PGD is based on a cognitive-behavioural framework that postulates that symptoms of acute grief persist and exacerbate to the point of impairment in people with PGD [45]. At least three processes influence this: (1) knowledge about the irreversibility of the separation is insufficiently integrated into representational knowledge about the self and the relationship with the lost person, which maintain separation distress and proximity-seeking responses; (2) a propensity to engage in persistent negative thinking about oneself, life and in one’s ability to deal with the pain and grief; and (3) a propensity toward fear and avoidance of external and internal reminders of the loss (termed “anxious avoidance”) and to withdraw from normal routines and valued activities driven by thoughts that one is unable to carry out and/or to enjoy such activities (termed “depressive avoidance”). Alleviation of PGD can be achieved by targeting these processes and using conventional cognitive-behavioural interventions. Imagery exposure (telling the story of the loss event, zooming in on the most painful aspects), *in vivo* exposure (visiting the scene of the death) and confrontational writing (writing a letter to the lost person, explaining what is missed most) are used to promote integration of the irreversibility of the loss with other knowledge. Socratic questioning (that is, identifying and discussing the validity and utility of maladaptive thoughts and altering those) and behavioural experiments (that is, specific assignments to test the validity of cognitions) are used to mitigate maladaptive thinking [46]. Behavioural assignments and skill-training are applied to replace maladaptive coping with more helpful ways of coping; for instance, exposure to avoided stimuli is used to target anxious avoidance, and behavioural activation is employed to interrupt the vicious cycle of depressive avoidance. Cognitive-behavioural Grief-Help therapy includes all these interventions, which are used in a simplified manner in accordance with the developmental level and cognitive abilities of the children.

The treatment is divided into five main parts, all of which are described in a workbook children use throughout treatment. In the first part of treatment (titled “Who died?”), the child is invited to talk about facts of the loss and things she or he misses and wished he or she could still share with the lost person. An important aim of this part is to encourage confrontation with the reality and pain of the loss for the client, as well as for the therapist to gather information about maladaptive thinking and behavioural patterns that are to be addressed later on in treatment. In the second part of the treatment (titled “What is grief?”) a task model (comparable to Worden’s [47] task model of grief) is introduced. The model explains four tasks bereaved children face in coming to terms with loss and the processes that may block achievement of these tasks: Task 1: Facing the reality and pain of the loss; Task 2: Regaining confidence in yourself, other people, life and the future; Task 3: Focusing on your own problems and not only those of others; and Task 4: Continuing activities that you used to enjoy. The task model provides a framework for interventions applied in the next stages of the treatment. For instance, in the third part (“Cognitive Restructuring”) cognitive restructuring is used to work on Task 2. In the fourth part of the treatment (titled “Maladaptive Behaviours”), graded exposure is used to work on Task 1, problem-solving skills are taught to address Task 3 and behavioural activation is used to help achievement of Task 4. In the fifth and final part of treatment (“Moving Forward after Loss”), the skills that are learned during the treatment are reviewed, summarized and written down. Additionally, a plan is discussed for continued practice of learned skills. Specific attention is paid to what the child could do, should his or her emotional problems become exacerbated. Moreover, during the course of treatment, the child writes three letters to an imaginary or real friend as a means by which to facilitate consolidation of the learning process and form a document of learned skills that can be consulted after treatment.

Children receive nine individual sessions. Surviving parents or caregivers receive five parental counselling sessions that are planned in parallel and aimed at supporting them in coaching their child during therapy. Therefore, the emotional problems of the parents are not the specific focus of these sessions.

The therapist reviews the child’s workbook together with the parents during the first two parental counselling sessions. The therapist and parents discuss the grief tasks the child is facing, based on the workbook parts “What is Grief?”, “Cognitive Restructuring” and “Maladaptive Behaviors”. They do this in a general manner but also focus specifically on patterns of behaviour and maladaptive thinking that may block the child’s grieving process.

To promote positive parenting skills and to strengthen the parent–child relationship, parents are given assignments to spend more quality time with their child and to improve communication skills. In sessions 3 and 4, there is a further focus on what parents can do to support their children in their grieving process by helping them to change maladaptive thoughts and behaviours (for example, by helping their children write cognitive diaries, supporting them during their exposure (i.e., to situations, persons and/or memories related to the deceased), solving problems, activating behaviours and providing rewards), and improvement of the parent–child relationship is further discussed. Session 5 is centred on relapse prevention, specifically focusing on signs that could signal to parents that their child might be experiencing new or increased intensity of problems. Parents are then encouraged to make a relapse prevention plan, and attention is again paid to maintaining a good parent–child relationship after the completion of treatment.

### Supportive counselling

Supportive counselling for childhood PGD is based on nondirective treatments for bereaved children [48,49] and adults [44,50] and on treatments for children with PTSD [51]. As a rationale for supportive counselling, it is explained to children that PGD coincides mostly with various emotional, social and practical difficulties and that discussing these could bring relief from the emotional burden of bereavement. Children are encouraged to express all their feelings and thoughts about the loss. The rationale for this encouragement of children to express their feelings is that bereaved children can experience many intense and different feelings and thoughts about the loss and that they can learn to cope with those feelings by expressing them. Expressing feelings of grief can take the form of talking, playing or making a memory box or book. Therapists are unconditionally supportive of issues children bring up and their attempts at problem-solving. They do not address cognitions and give no instructions for exposure.

As in Grief-Help therapy, supportive counselling includes nine individual sessions with the child and five counselling sessions with the parents or other caregivers. The treatment is divided into three parts. The first sessions are devoted to identifying difficulties children experience in their everyday lives. Children are encouraged to express all their feelings and thoughts about the loss in any way they like. As a second step, the therapist and child review all themes that have been identified in the first phase of therapy in more detail. The child decides if she or he prefers to talk, play or express their feelings in any other possible way. In the last phase, the therapist and child speak about or play saying goodbye to each other.

Counselling sessions with parents are planned every 2 weeks. In the first session, a plan is made about which themes parents want to discuss and in which order they should be talked about. The therapist helps the parents to think about solutions to problems that they encounter in supporting their child. The therapist can also suggest themes. Examples include (1) the grieving process; that is, What does the parent think about the grieving process, how do parents support their child, what are the main problems for the child? (2) feelings and thoughts the child expresses about the loss and the reactions of the parents to these expressions; (3) coping behaviours of the child related to the loss; that is In what ways does the child cope with his or her feelings and thoughts? What do parents think helps the child and what does not? and (4) the development of the child in the near future; that is, What are potential problems, given the loss and the development of the child thus far? How do the parents cope with those ideas about the future of the child? There are no homework assignments for children and parents in the supportive counselling sessions.

### Statistical analyses

Most analyses will be conducted using the software program SPSS (SPSS, Inc, Chicago, IL, USA). Descriptive analyses will be carried out using standard methods. For analysis of the primary and secondary outcome measures, we will use analysis of covariance with the outcome measures posttreatment or at follow-up sessions as dependent variables, treatment condition as a factor and preintervention scores of the outcome variables as covariates. We will compare the scores of children in both groups with their own scores on previous assessments by using regression analysis and/or structural equation modelling. Mediator and moderator models will be tested. Analysis and reporting of the results will be carried out according to the CONsolidated Standards of Reporting Trials (CONSORT) 2010 Statement guidelines [52].

### Protection of data privacy

Children and parents participating in this study will be assigned a number. This number will be used in the data set. Key lists of participants’ names and assigned numbers will be stored separately from the data in lockable cabinets and rooms and will be deleted after final data analyses. No conclusions will be drawn from the data on individual clients.

### Publication policy

We plan to publish the results of this study in peer-reviewed national and international journals. The results will be presented at national and international scientific conferences.

## Discussion

There is growing evidence that childhood PGD is a clinically significant condition [3,7]. However, there is limited knowledge about effective treatment interventions for children confronted with loss [11]. Given the lack of effective treatments for childhood PGD, research is urgently needed. Herein we have presented the protocol of a study designed to investigate the effectiveness of a novel cognitive-behavioural treatment called *Grief-Help* in comparison with supportive counselling for childhood PGD and to enhance knowledge about the variables mediating and moderating the effects of Grief-Help therapy. The current study is a randomised controlled trial among bereaved children and adolescents (ages 8 to 18 years) with elevated PGD who will be randomly allocated to one of the intervention groups. To the best of our knowledge, we are the first researchers to compare Grief-Help therapy with supportive counselling in bereaved children. In this study, we hope to contribute to the treatment of childhood PGD as well as to knowledge about mediators and moderators of change.

This study has several strengths. First, we will compare two different treatment conditions instead of a treatment and a waiting list condition. Both treatments are similar in certain aspects. Both treatments comprise nine sessions for the children and five sessions for the parents, and, in both conditions, children and parents will get the same psychoeducation. This will make it possible to evaluate different outcomes in terms of specific interventions, such as cognitive restructuring, exposure (both imaginary and *in vivo*), behavioural activation and confrontational writing. The second strength of this study is the fact that we will use validated measures. A third strength is that there will be multiple sources of information, because both parents and children will be administered multiple measures. Another strength is that, in this study, we will go beyond simple effectiveness questions and examine mechanisms that can explain the effects (for example, positive parenting, maladaptive cognitions and behaviours) and possibly moderate them (for example, demographic variables, time since loss, cause of death). By doing this, we will gain better insight into what works for whom.

The study also has potential weaknesses. First, it is possible that therapists (or someone else who does the intake interview in a specific setting) will be biased toward selecting children who are likely to benefit from treatment instead of including in A1 all children who meet the inclusion criteria. Second, only family members who are participants and receivers of treatment will be used as informants, without including more objective persons such as teachers.

## Trial status

Ethical approval for this study has been obtained from a medical ethics committee (Central Committee on Research Involving Human Subjects NL30528.041.09). All therapists have been trained at the different participating sites. Patient recruitment is ongoing and will continue until mid-2014.

## Competing interests

The authors declare that they have no competing interests.

## Authors’ contributions

PB, MD, PP, JvdB and MS jointly obtained funding for the study. All authors contributed to the study design. MS coordinates recruitment of the participants and data collection during the study. PB, MD and PP will supervise the process. MS wrote the manuscript in close collaboration with the other authors. All authors read and approved the final manuscript.
